# Low temperature *a*/*b* nanotwins in Ni_50_Mn_25+*x*_Ga_25−*x*_ Heusler alloys

**DOI:** 10.1038/s41598-018-30388-8

**Published:** 2018-08-09

**Authors:** L. Straka, J. Drahokoupil, P. Veřtát, M. Zelený, J. Kopeček, A. Sozinov, O. Heczko

**Affiliations:** 1 Institute of Physics, Czech Academy of Sciences, Na Slovance 2, 182 21, Prague, Czech Republic; 2 Faculty of Mathematics and Physics, Charles University in Prague, Ke Karlovu 5, 12116 Prague, Czech Republic; 3 Institute of Materials Science and Engineering, NETME Centre, Faculty of Mechanical Engineering, Brno University of Technology, Technická 2896/2, 616 69, Brno, Czech Republic; 4 Lappeenranta University of Technology, Material Physics Laboratory, Laitaatsillantie 3, Savonlinna, 57170 Finland

## Abstract

We have found low temperature *a*/*b* nanotwins having (110) twinning plane in a five-layered modulated martensite phase of Ni_50_Mn_25+*x*_Ga_25−*x*_ (at. %) Heusler alloys and identified the particular region in phase diagram where the nanotwinning occurs. Evolution of the structure with decreasing temperature was studied by X-ray diffraction using single crystals exhibiting magnetic shape memory effect. The merging of (400) and (040) lines upon cooling for 2.6 < *x* < 3.5 indicated *a*/*b* nanotwinning originating from the refinement of initially coarse *a*/*b* twins. Refinement of the twins with decreasing temperature was observed directly using scanning electron microscopy. The prerequisite for nanotwinning is an extremely low twin boundary energy, which we estimated using first-principles calculations to be 0.16 meV/Å^2^. As the nanotwinning distorts the relation between the crystal lattice and the X-ray diffraction pattern, it should be taken into consideration in structural studies of Ni-Mn-Ga Heusler alloys.

## Introduction

The Ni_2_MnGa system has a prominent position among Heusler alloys^1^, as it is only one of the very few materials which exhibit a so-called *giant magnetic field-induced strain* or *magnetic shape memory* (MSM) *effect* in a moderate magnetic field (<1 T)^2–5^. The effect known also as *magnetically-induced reorientation* (MIR) is closely related to the high mobility of twin boundaries within the martensite structure of the material and thus the understanding of martensite structure and microstructure is crucial. The structure of alloys close to Ni_2_MnGa composition has been studied very intensively during recent decades but there are still some controversies, particularly about the nature of the lattice modulation^6–10^.

The structural transition to a modulated martensite was reported by Webster^11^. Martynov and Kokorin^12^ identified three major types of martensites in single crystals close to Ni_2_MnGa composition: non-modulated tetragonal martensite (NM) and five- (10 M) and seven-layered (14 M) modulated martensites with nearly harmonic shear displacements along $\mathrm{(110)[1}\bar{1}\mathrm{0]}$ system. X-ray, electron, and neutron diffraction studies such as that of Righi *et al*.^7,13,14^, Fukuda *et al*.^15^, and Kushida *et al*.^16^, respectively, indicated incommensurate lattice modulation in Ni_2_MnGa^10^. Kaufmann *et al*.^17–19^ following Khachaturyan’s work^20^ suggested that modulated martensite structures in Ni-Mn-Ga can be explained by the adaptive martensite theory. In this approach, the modulated martensites are considered as nanotwinned form of the original non-modulated tetragonal martensite. Such nanotwins are two, three, or five atomic planes thick (i.e. $\le 1$ nm) and will be referred to further on as *adaptive nanotwins*. The difference between nearly harmonic lattice modulation and nanotwinning is not large and it may be beyond the capabilities of experimental methods to distinguish between the two^9,21^. Here we would like to point out that the adaptive martensite theory and incommensurate lattice modulation are in contradiction as the adaptive nanotwins cannot be formed from a non-integer number of unit cells. Apparent incommensurality can be seen when stacking faults are inserted into adaptive martensite^22^.

The lattice of 10 M martensite is monoclinic but very close to tetragonal (*a* ≈ *b*, *γ* ≈ 90°)^23^. The nearly equal *a* and *b* axes are not equivalent crystallographically but are nearly equivalent from the point of view of twinning. For that reason we further use {) notation, which means that the first two indexes are permuted while the third one remains constant, e.g. {110) means four possible planes (110), (1$$\bar{1}$$0), ($$\bar{1}$$10), and ($$\bar{1}$$$$\bar{1}$$0).

Seiner *et al*.^24^ pointed out based on experimental results and theoretical calculation that the 10 M martensite exhibits a deep four-level twinning hierarchy. On macro- and mesoscale, the typically observed twins are so-called *a*/*c twins* of Type 1 or Type 2 with {101) or approx. {10 1 10) twinning plane, respectively. The compound {100) twins or *modulation twins* are typically seen on mesoscale as internal twins within *a*/*c* twins. The modulation twins are internally twinned further by the so-called *a*/*b twins*^23^, which are roughly around a micrometer or smaller scale^25,26^. The *a*/*b* twins are compound twins with twinning plane {110). They occur due to difference between the lattice parameters *a* and *b*, which is very small but not negligible. They can refine to very small size (<20 nm) near the martensite transformation owing to the presence of an austenite nucleus^27^. In the adaptive martensite concept, the *a*/*b* twins are internally twinned by the adaptive nanotwins forming a modulated structure.

In general, the nanotwinning is not uncommon and is often reported in ferroelectrics^28–33^. Although the ferroelectrics are oxides, we can use the theoretical apparatus and observations related to nanotwinning also for magnetic shape memory alloys. The theory of diffraction from a nanotwin superlattice has been developed by Wang *et al*.^34–36^. The important consequence of nanotwinning is that it generates peak shifts and symmetries in the diffraction pattern, which do not correspond to the true unit cell. It has been clearly illustrated in experiments that some higher symmetry phases are superficial originating from (adaptive) nanotwinning of the original lower symmetry phase^31–33^. Previously, Ustinov *et al*.^37^ calculated the peculiar diffraction effects and apparent lattice symmetry resulting from the short range order of adaptive nanotwins in Ni-Mn-Ga. Straka *et al*.^27^ reported experimental observations of *a*/*b* nanotwins in a very narrow (<1 K) temperature range in close vicinity of martensitic transformation. Their origin was ascribed to the branching at the austenite-martensite interface.

In this article we report on our finding of *a*/*b* nanotwinning at low temperatures in Ni_50_Mn_25+*x*_Ga_25−*x*_ alloys. These particular compositions are of strong interest for being good candidates for practical applications of MIR due to low twinning stress and relatively high martensite transformation temperature^38^. In the paper we first demonstrate the peculiar diffraction effects related to *a/b* nanotwinning, then we confirm the twin refinement in a scanning electron microscope (SEM), and finally we identify the particular region in a phase diagram of Ni-Mn-Ga where the nanotwinning occurs. Additionally, we present total energy calculations related to *a*/*b* nanotwinning and discuss its potential origin and impact on structure determination.

## Results

### Theoretical diffraction pattern from a nanotwinned lattice

**Figure 1:**
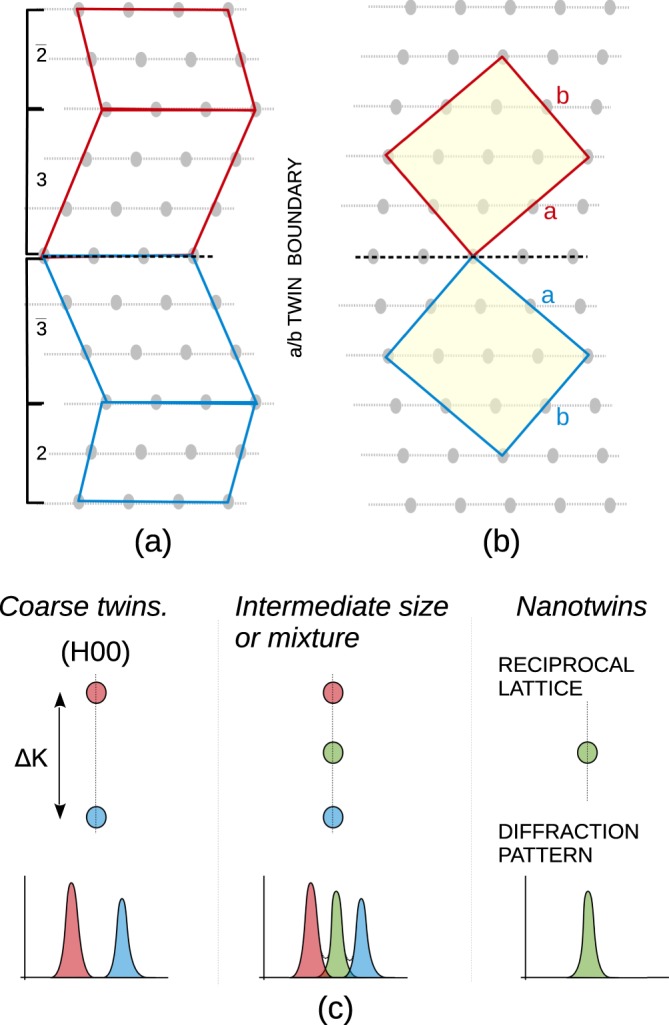
**a**) *a*/*b* twins formed as an inversion of ${\mathrm{(3}\bar{2})}_{2}$ stacking sequence. **b**) Corresponding average lattice and unit cell with *a* and *b* axes and twinning plane marked. **c**) Reciprocal space points and diffraction pattern related to (H00) lines in twinned and nanotwinned material^34,35^.

Using the adaptive martensite concept, the 10 M martensite is built from the adaptive nanotwins of a non-modulated martensite, following the ${\mathrm{(3}\bar{2})}_{2}$ stacking sequence. The *a*/*b* twin can then be seen as the sequence inversion, i.e., one twin domain is formed by ${\mathrm{(3}\bar{2})}_{2}$ sequence and the corresponding mirror twin domain by ${(\bar{3}\mathrm{2)}}_{2}$ sequence, Fig. 1a^22,27,39^. For the sake of clarity, the figure does not show the ordering of atoms, which requires repeating the $\mathrm{(3}\bar{2})$ sequence twice. To further reduce the complexity, we draw the *a*/*b* twin in an *average lattice*, using coordinates derived from the original cubic L2_1_ cell, Fig. 1b. For the study of principal reflections, which is presented here, this simplification suffices; naturally, for studying superstructure reflections it would be necessary to include the modulation of lattice or the adaptive nanotwinning concept.

The expected effects of twinning and nanotwinning on the diffraction pattern according to the theory^34,35^ are illustrated in Fig. 1c. The twinning results in splitting of (H00) principal spots in a reciprocal space along the direction perpendicular to the twinning plane. This effect can be alternatively seen as a simultaneous observation of two nearby spots: (H00) from one twin domain and (0H0) from the other twin domain, which is rotated by twinning to close vicinity of (H00). In a nanotwinned material, these two spots disappear and a nanotwin superlattice spot appears instead, with the exact position in between the original spots given by individual variant fractions and lever rule. For combined twinning and nanotwinning the split (H00) and the nanotwin superlattice spot can be observed simultaneously, resulting in a triple peak. For quantitative results of theoretical calculation in the Ni_50.0_Mn_28.7_Ga_21.3_ 10 M martensite, see ref.^27^.

### Observed effect of mosaicity vs. effect of twinning

**Figure 2:**
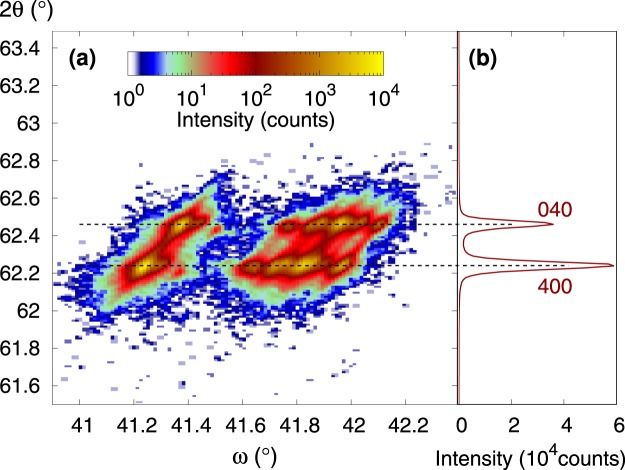
The 2*θ*−*ω* map in the Ni_50.0_Mn_28.2_Ga_21.8_ single crystal at 295 K (**a**) and the map intensity integrated over *ω* shown as a function of 2*θ* (**b**). (The original measurement was performed with Co X-ray tube but 2*θ* was recalculated for Cu tube to enable direct comparison with other figures in the article).

The mosaicity of real single crystal can result in a diffraction peak splitting, which can be confused with the splitting originating from twinning. Thus the mosaicity must be carefully considered in experiment. The high resolution 2*θ* − *ω* map of the Ni_50.0_Mn_28.2_Ga_21.8_ single crystal at room temperature is shown in Fig. 2a. The mosaicity is to be seen as a peak splitting along *ω* and this is what we also observe in the figure. The two major peaks are each split into about five sub-peaks along *ω*. From the position of side sub-peaks the misorientation of low angle grains is about one degree. The split into two major peaks along an approximately vertical direction is due to *a*/*b* twinning. Clearly, the splits due to mosaicity and twinning are independent and can be easily separated. The separation is illustrated in Fig. 2b, where the intensity is integrated over *ω* angle. In following experiments with 2*θ* − *ω* scans, one can imagine that the map is sampled along 2*θ* with partial integration over *ω* thus the split caused by twinning is clearly distinguished as a double (400)-(040) peak. It is obvious that while mosaicity can contribute to apparent change of the (400) or (040) line intensity, e.g. due to geometrical misalignment, it cannot result in the appearance of an extra central line observed in the following experiments.

### Development of the diffraction pattern with temperature

**Figure 3:**
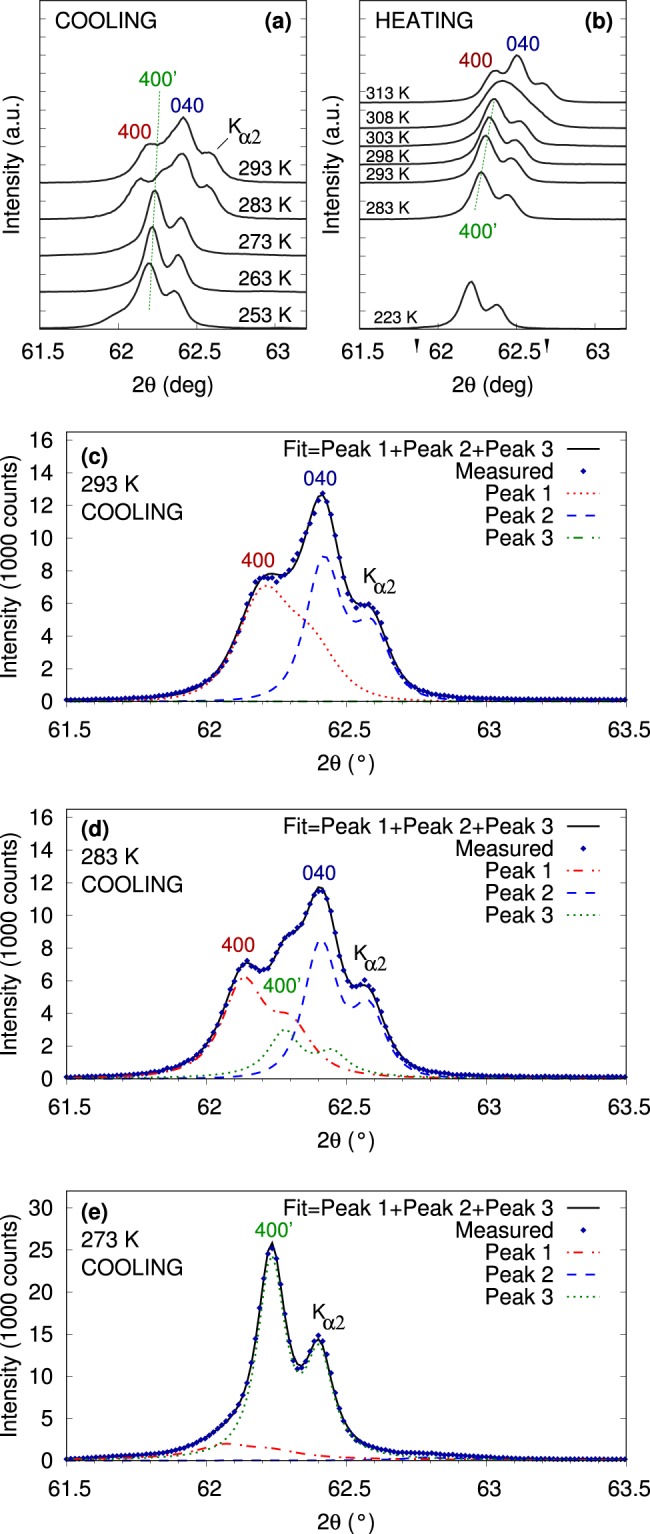
The 2*θ*−*ω* scans in the selected 2*θ* interval performed during quasistatic cooling (**a**) and heating (**b**) of the Ni_50.0_Mn_28.2_Ga_21.8_ single crystal. (400) and (040) lines of 10 M phase and (400)’ line of 10 M’ phase are marked. **c**,**d**,**e**) Decomposition of the observed profile into individual lines at selected temperatures during cooling.

The 2*θ*−*ω* scans in the Ni_50.0_Mn_28.2_Ga_21.8_ single crystal as a function of temperature are shown in Fig. 3a (cooling) and Fig. 3b (heating). The selected range of 2*θ* corresponds to (400) and (040) reflections due to twinning, Fig. 1c. The ratio of peak intensities corresponding to twin variant ratio is different from Fig. 2b using a different sample with different thermomechanical history. Importantly, significant changes in peak profiles are clearly seen during cooling and heating: the (400)-(040) double peak changes to a single peak at 273 K during cooling and, conversely, this single peak separates into two lines at 313 K during subsequent heating.

A more detailed analysis is shown in Fig. 3c,d,e for selected temperatures with individual peaks decomposed. The two peaks observed at 293 K correspond to (400) and (040) lines. At 283 K, a third extra peak appears. This extra line is marked as (400)’ for the purpose of further discussion. At 273 K and below, the (400)’ line dominates the pattern. The line disappears after reheating to 313 K and is replaced by the original (400)-(040) double peak.

**Figure 4:**
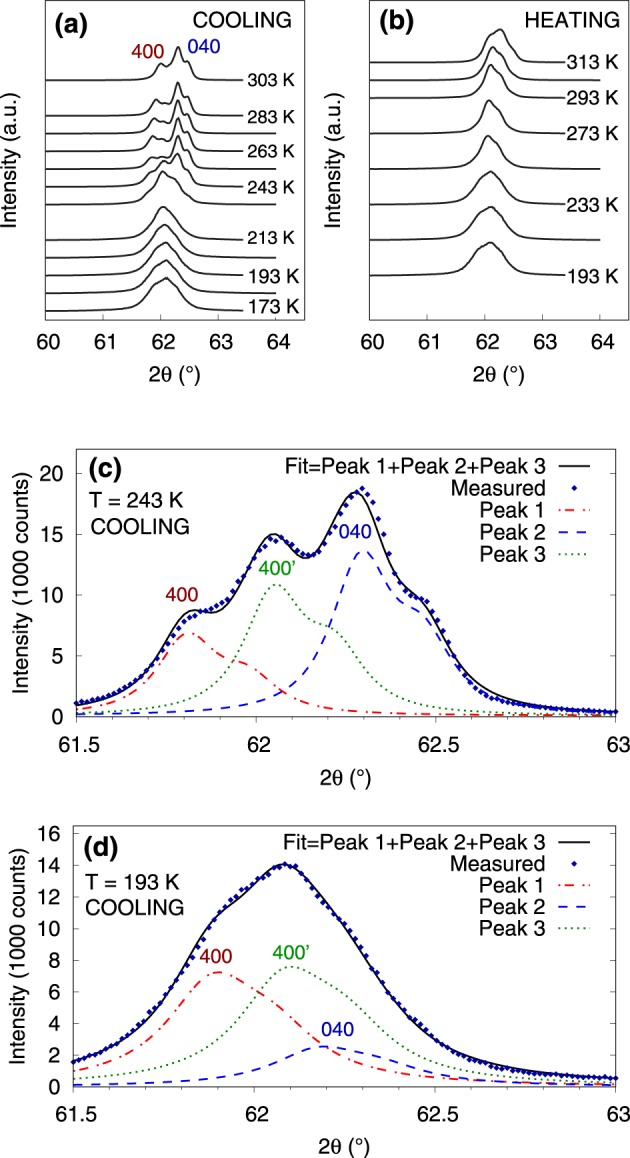
The 2*θ*−*ω* scans in the selected 2*θ* interval performed during quasistatic cooling (**a**) and heating (**b**) of the Ni_50.0_Mn_28.5_Ga_21.5_ single crystal. (400) and (040) lines are marked. **c**,**d**) Decomposition of the observed profile into individual lines at selected temperatures during cooling.

Similar development of (400)-(040) double peak with temperature is observed in the Ni_50.0_Mn_28.5_Ga_21.5_ single crystal, Fig. 4. Upon cooling, Fig. 4a, the (400)-(040) double peak changes substantially at 243 K transforming into a single broad peak. Upon heating, Fig. 4b, the re-splitting into (400) and (040) occurs around 313 K. Moreover, the diffraction pattern after reheating exhibits different peak intensities indicating different ratio of *a* and *b* variants as compared to initial state. Due to the comparable intensity and *a* and *b* being very close to each other the lines are not distinguished in the profile directly but only using a detailed analysis. The detailed analysis for cooling in Fig. 4c,d shows that–in contrast to Ni_50.0_Mn_28.2_Ga_21.8_–the (400)’ line appears alongside the (400) and (040) and all the lines simultaneously maintain a substantial intensity down to 173 K.

The observed common changes in the diffraction pattern related to (400) and (040) principal reflections indicate *a*/*b* nanotwinning at low temperatures. A new (400)’ central line replaces or accompanies the original (400)-(040) double peak, which exactly corresponds to the expected effect of nanotwinning on the diffraction pattern; compare Figs 3c,d,e and 4c,d with Fig. 1c. While the dominant single (400)’ line for Ni_50.0_Mn_28.2_Ga_21.8_ corresponds to dominant *a*/*b* nanotwinning (Fig. 3), presence of the peak triplet for Ni_50.0_Mn_28.5_Ga_21.5_ suggests an intermediate state or a mixture of coarse twins and nanotwins (Fig. 4). Considering the prior case with only a single (400)’ line and the adaptive diffraction condition^34,35^ $m < 2/sH$ fulfilled, where *s* = 0.0045 is twinning shear and *H* = 4 is reciprocal space coordinate, the corresponding size of nanotwins *m* is determined to be less than 17 nm^27^.

### Confirmation of twin refinement by SEM

**Figure 5:**
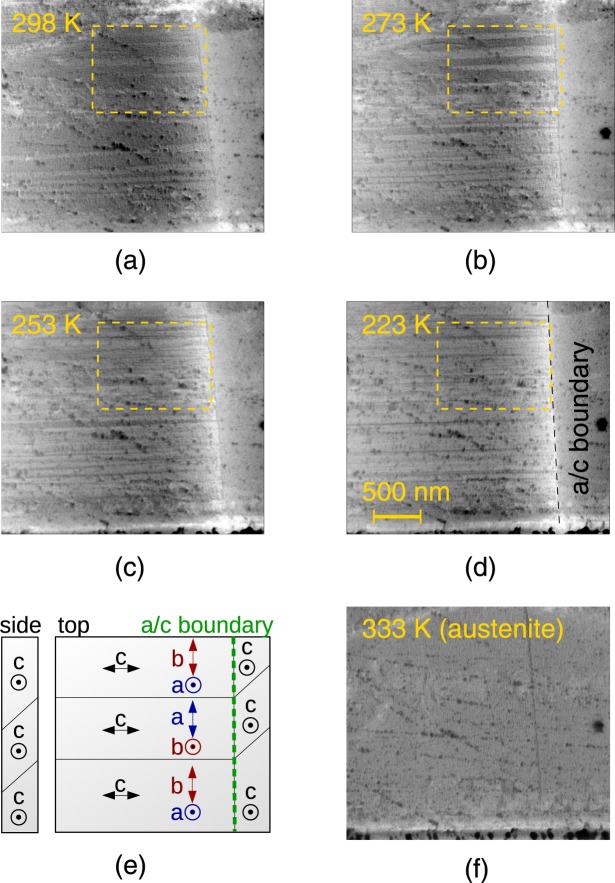
**a**–**d**) Temperature development of horizontal band contrast related to *a*/*b* twinning obtained using BSE in SEM in the Ni_50.0_Mn_28.2_Ga_21.8_ single crystal. Dashed frame guides to the region where the changes are best seen. Gamma (contrast) and brightness were adjusted in acquired figures to better expose the weak horizontal band contrast. See supplementary information for original nonadjusted full size micrographs. **e**) Crystal orientation and interpretation of the observed microstructure. **f**) Observation in austenite with no band contrast.

The X-ray diffraction measurements suggest that the *a*/*b* nanotwins occur at low temperatures. To confirm or reject this hypothesis we performed direct observation of the *a*/*b* twin laminate in SEM. It has been shown recently that the *a*/*b* twinning can be observed using a back-scattered electrons (BSE) contrast in SEM^26^ and we applied the same method here. The micrographs obtained using the BSE contrast at selected temperatures are shown in Fig. 5. The observations with decreasing temperature were made near {101) twin boundary. The crystal was oriented as illustrated schematically in Fig. 5e: the vertical contrast corresponded to {101) twinning (single *a*/*c* twin boundary) while the horizontal contrast corresponded to the *a*/*b* twinning.

The *a*/*b* twins near the {101) boundary are in hundreds nm scale at 298 K, Fig. 5a. After cooling to 273 K, Fig. 5b, we observe a mixture of twins with about the same size and of much finer twins (bottom part of the micrograph). At 253 K the majority of the *a*/*b* twins consists of fine twins in the scale of ten(s) of nm. Further cooling to 223 K indicates progressing refinement. The reference observation after heating to austenite with no horizontal nor vertical contrast is shown in Fig. 5f. It confirms that the contrast observed in Fig. 5a,b,c,d is related to twinning, as it occurs only in the martensitic phase.

In conclusion, the SEM observations, Fig. 5, show clearly the gradual refinement of twins down to the scale of tens of nm with decreasing temperature. Combined with the described changes in the diffraction pattern and calculation^27^, we can confidently claim that low temperature *a*/*b* nanotwinning occurs in the studied alloys.

### Region of *a*/*b* nanotwinning in phase diagram

**Figure 6:**
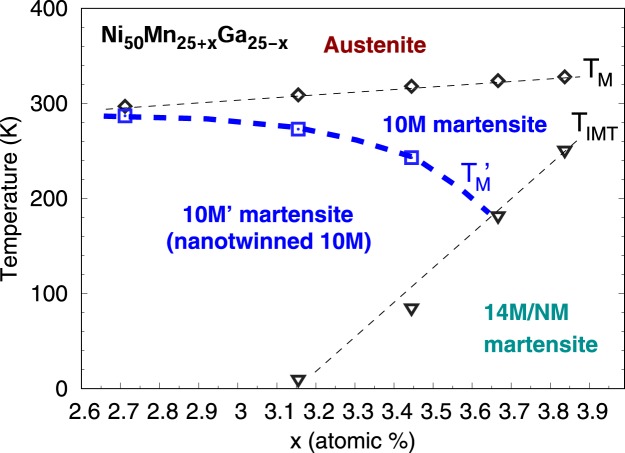
Phase diagram with the region of *a*/*b* nanotwinned martensite (10 M’) marked. Temperatures marked: *T*_M_ ≈ *M*_S_ ≈ *M*_F_ - transformation to martensite (10 M) upon cooling, ${T}_{{\rm{M}}}^{\prime}$ - transformation to nanotwinned martensite (10 M’) upon cooling, *T*_IMT_ - intermartensite transformation upon cooling.

Phase diagram was constructed using martensite (${T}_{{\rm{M}}}\approx {M}_{S}\approx {M}_{f}$) and intermartensite (*T*_*IMT*_) transformation temperatures^38^. Based on the measurement of five alloys with Mn excess *x* between 2.6 and 3.9 atomic %, the region of *a*/*b* nanotwinning is marked in the phase diagram as 10 M’, Fig. 6. There is essentially no nanotwinning at room temperature for alloys with $x > 3.5$ and thus the ordinary 10 M phase is observed. However, for alloys with $2.6 < x < 3.5$ the *a*/*b* nanotwinning occurs upon cooling below ${T}_{{\rm{M}}}^{\prime}$. Note that this transition is in reality gradual and not sharp (Fig. 5), the ${T}_{{\rm{M}}}^{\prime}$ and the sharp line correspond only to the sharp change in the diffraction pattern we observe, when crossing the adaptive diffraction condition (Figs 3 and 4)^34,35^. The reverse transition from nanotwins to coarse twins occurs upon heating with thermal hysteresis in transformation of up to few tens of kelvins. The reverse transition is not marked in the diagram for the sake of clarity.

The *a*/*b* nanotwinning has previously been found in Ni-Mn-Ga single crystals in a very narrow temperature range (<1 K) very near (reverse) the martensitic transformation and has been ascribed to the presence of an austenitic nucleus. The newly found region of nanotwinning as indicated in the phase diagram occurs at low temperatures in a much broader temperature range. It cannot be caused by the appearance of an austenitic nucleus, since it occurs on cooling and far away from the martensitic transformation temperature.

The location and shape of the discovered region of *a*/*b* nanotwinning suggests strongly that also alloys with $x < 2.6$ can inherently be nanotwinned. That concerns especially the particular case of stoichiometry *x* = 0, i.e., Ni_50_Mn_25_Ga_25_ or equally Ni_2_MnGa.

### *Ab initio* calculation of *a*/*b* twin boundary energy

The prerequisite for *a*/*b* nanotwinning in a 10 M martensite is an extremely low energy of *a*/*b* twin boundary, *σ*_*a*/*b*_. The *ab initio* calculations based on density functional theory (DFT) were employed to estimate the *σ*_*a*/*b*_ in stoichiometric Ni_2_MnGa. In the framework of DFT the twin boundary energy in *a*/*b* twins can be calculated as:

1 $${\sigma }_{a/b}=\frac{{E}_{a/b}-{E}_{10{\rm{M}}}}{n\cdot A},$$

where *E*_*a*/*b*_ is the total energy of a supercell containing the *a*/*b* twins, *E*_10M_ is the total energy of a supercell of a perfect 10 M lattice with the same number of atoms, *n* is the number of *a*/*b* twin boundaries in the supercell and *A* is twin boundary area within the supercell. From the adaptive martensite concept it follows that the *a*/*b* twins are formed by ${\mathrm{(3}\bar{2})}_{2}$ stacking sequence inversion. There can be two possible orderings of atomic planes at the *a/b* twin boundary (indicated by “|”): $\mathrm{...3}\bar{2}\mathrm{|2}\bar{3}\mathrm{...}$ and $\mathrm{...}\bar{2}\mathrm{3|}\bar{3}\mathrm{2...}$. Our supercell always contains the same number of both types of ordering, which is necessary for keeping the orthorhombic symmetry of the lattice, periodic boundary conditions, and a reasonable number of atoms comparable with the perfect supercell. Thus, the calculated value can be viewed as an average energy of both types and in reality the twin boundary energy could be even smaller if one type of ordering is energetically preferred.

**Figure 7:**
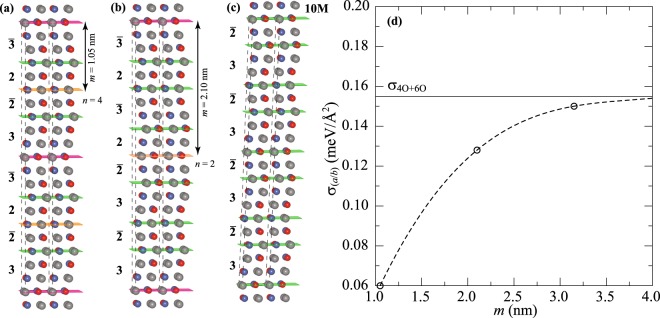
Atomic arrangements corresponding to *a*/*b* nanotwinned 10 M structure (gray balls - Ni, blue and red balls - Mn and Ga): **a**) ${\mathrm{(3}\bar{2}\mathrm{|2}\bar{3})}_{1}$ stacking, **b**) ${\mathrm{(3}\bar{2}3\bar{2}\mathrm{|2}\bar{3}2\bar{3})}_{1}$ stacking. **c**) Ideal 10 M structure with ${\mathrm{(3}\bar{2})}_{2}$ stacking. The computational 1 × 1 × 2 (a), 1 × 1 × 1 (b) and 1 × 1 × 2 (c) supercells are marked by dashed lines. Green planes correspond to adaptive nanotwin boundaries. Orange and pink planes represent $\mathrm{...3}\bar{2}\mathrm{|2}\bar{3}\mathrm{...}$ and $\mathrm{...}\bar{2}\mathrm{3|}\bar{3}\mathrm{2...}$ types of *a*/*b* twin boundaries, respectively. **d**) Calculated *a*/*b* twin boundary energy *σ*_*a*/*b*_ as a function of twin size *m*. Solid horizontal line represents average energy of adaptive nanotwin boundaries in 4 O and 6 O structures. Dashed line is only a guide for the eye.

The experimentally observed size of the *a*/*b* twins is around 10 nm. Because it is impossible to create a model of such twins for DFT simulations, we determine the energy by converging *σ*_*a*/*b*_ with respect to increasing twin width *m* for 1.05 nm, 2.10 nm, and 3.15 nm. These twin widths correspond to ${\mathrm{(3}\bar{2}\mathrm{|2}\bar{3}|)}_{1}$ (Fig. 7a), ${\mathrm{(3}\bar{2}3\bar{2}\mathrm{|2}\bar{3}2\bar{3}|)}_{1}$ (Fig. 7b), and ${\mathrm{(3}\bar{2}3\bar{2}3\bar{2}\mathrm{|2}\bar{3}2\bar{3}2\bar{3}|)}_{1}$ (not shown) stacking sequences described by supercells containing a twin boundary of both types ($\bar{2}\mathrm{|2}$ and $\bar{3}\mathrm{|3}$) and 40, 80, and 120 atoms, respectively. The twin width *m* = 1.05 nm corresponds to the smallest possible twin. Total energies of nanotwinned supercells were compared with 1 × 1 × 1, 1 × 1 × 2, and 1 × 1 × 3 supercells of a perfect 10 M lattice described by ${\mathrm{(3}\bar{2})}_{2}$ stacking sequence (Fig. 7c). To ensure that our calculations are not influenced by changing the supercell size, we calculated *σ*_*a*/*b*_ for the smallest ${\mathrm{(3}\bar{2}\mathrm{|2}\bar{3}|)}_{1}$ twins also in 1 × 1 × 2 and 1 × 1 × 3 supercells, which contained 4 and 6 *a/b* nanotwin boundaries, and found no significant differences.

The calculated twin boundary energies *σ*_*a*/*b*_ as a function of twin size *m* are displayed in Fig. 7d. The energy increases with increasing *m* from the lowest value *σ*_*a*/*b*_ = 0.06 meV/Å^2^ corresponding to the smallest twin size of 1.05 nm. For enlarging twin size the energy converges to a constant value which is not influenced by the interaction of adjacent twin boundaries as they are far apart. The energy is comparable with a value ${\sigma }_{4{\rm{O}}+6{\rm{O}}}=({\sigma }_{4{\rm{O}}}+{\sigma }_{6{\rm{O}}})/{\rm{2}}\dot{=}0.16$ meV/Å^2^ (horizontal line in Fig. 7d), which is an average twin boundary energy of adaptive nanotwins forming a hypothetical 4 O martensite with only $\bar{2}\mathrm{|2}$ boundaries (*σ*_4O_ = −0.67 meV/Å^2^) and 6 O martensite with only $\bar{3}\mathrm{|3}$ boundaries (*σ*_6O_ = 0.98 meV/Å^2^). These structures represent the nanotwinning of a nonmodulated martensite with ${\mathrm{(2}\bar{2})}_{1}$ and ${\mathrm{(3}\bar{3})}_{1}$ stacking sequences, respectively^40^.

In summary the energy of *a*/*b* twin boundaries at 0 K converges to *σ*_*a*/*b*_ = 0.16 meV/Å^2^ for twin boundaries being far apart (>3 nm).

## Discussion

### Effect of *a*/*b* nanotwinning on structure determination

We found and experimentally confirmed the low temperature {110) or *a*/*b* nanotwins in Ni-Mn-Ga 10 M martensite. The general and the most serious impact of our finding is that the presence of nanotwinning distorts the usual direct relationship between the crystal lattice and the observed diffraction pattern. In other words the presence of nanotwinning superlattice can shift the principal reflections and generate false apparent symmetries of the lattice^34,35,37^. In our particular case the nanotwinning makes the lattice seemingly orthorhombic (for parallelogram with *a* = *b* the diagonals are perpendicular and *c* axis is perpendicular to *ab* plane^27^). In reality, however, the true unit cell remains monoclinic, albeit nanotwinned.

Previous studies indicated different modulated 10 M structures depending on excess Mn content. Particularly Righi *et al*.^7^ reported splitting of (400) line for high Mn content and no split for stoichiometric Ni_2_MnGa. Using Rietveld refinement of powder diffraction data they identified *monoclinic basic structure* in an Mn-rich alloy and *orthorhombic basic structure* for the stoichiometric composition. That agrees with present observations and the finding that some kind of transition exists with decreasing Mn content or decreasing temperature. For the present case with excess Mn, the transition is clearly of a nanotwinning character, Fig. 5.

The shape and location of the nanotwinning region in the phase diagram, Fig. 6, suggest that the structure of stoichiometric Ni_2_MnGa (*x* = 0) may actually also be a nanotwinned form of the original 10 M phase. This would result in the apparent orthorhombic symmetry. A reliable test of this hypothesis is, unfortunately, beyond the capabilities of our current experimental arrangement. The key differences between the present study and a stoichiometric alloy are: i) the transformation temperature is lower (≈200 K), ii) the reference microstructure with coarse twins allowing detection of transition to nanotwinning (Fig. 5) will not presumably be present at all during cooling, and iii) there is a pre-martensite transformation which may introduce significant differences and influence the final modulated martensite structure.

Marriager *et al*.^10^ considered the possibility that incommensurability of the lattice in Ni_2_MnGa (*x* = 0) is only apparent and caused by stacking faults in ${\mathrm{(3}\bar{2})}_{2}$ sequence. Based on comparison of the diffraction experiment and calculation of the structure with the stacking faults they rejected this possibility and concluded that the lattice was truly incommensurate and not nanotwinned at all (excluding also adaptive nanotwins). However, they considered only regular stacking faults and not the inverting stacking fault leading to *a*/*b* nanotwinning. Thus it seems that the question of the true structure of Ni_2_MnGa still remains open for future investigations.

The important question to ask is why should the nanotwinning occur at all. The driving force for the *a*/*b* nanotwinning may originate from the elastic incompatibility on *a*/*c* twin boundaries resulting in branching of *a*/*b* twins close to these boundaries^24^. This is a similar situation as described for the case of nanotwinning driven by twin branching near the phase interface with the presence of an austenitic nucleus^27^. Depending on the twin energy the branching near *a*/*c* boundary can extend over significant volume of the material effectively nanotwinning part of or whole sample.

A distinct and driving mechanism-independent prerequisite for the nanotwinning in a large volume is an extremely low energy of *a*/*b* twin boundary. *Ab initio* calculation suggests that the energy of *a*/*b* twin boundaries at 0 K is ${\sigma }_{(a/b)}\approx 0.16\,$ meV/Å^2^ or less in Ni_50_Mn_25_Ga_25_, which may further decrease in finite temperature. This energy is indeed extremely small in comparison to macroscopic mobile {101) twin boundaries with ${\sigma }_{(a/c)}\approx 1$ eV/Å^2^ or with the energy of adaptive nanotwins of about 1 meV/Å^2 ^^17,19,41^.

### Effect of *a*/*b* nanotwinning on martensitic transformation and magnetic shape memory properties

The 10 M’ nanotwinned phase was found previously only in a very narrow temperature interval between the austenite and martensite. It was suggested that this intermediate transitional phase could be regarded as an adaptive phase within the (adaptive) 10 M phase. The 10 M martensite (($3\bar{2}$)_2_ stacking sequence) is the product of adaptation to austenite on the habit plane on nanoscale (≈1 nm), while *a*/*b* nanotwinning (the inversion of the ($3\bar{2}$)_2_ stacking sequence) simultaneously provides the adaptation on the scale of tens of nanometers to the twinned martensitic structure on the other side of transitional region^27^. However, the narrow temperature window of stability and closeness of the martensitic transformation make systematic experimetal investigations difficult. Here we show that the stable 10 M’ phase can exist in a broad temperature range far below the martensitic transformation. That opens a good opportunity to apply a variety of experimetal methods in order to comprehend the properties of the nanotwinned phase in detail and then apply this understanding in order to fully identify the role of nanotwinning for martensitic transformation and formation mechanism of 10 M adaptive martensite^17,19,20,28,29,39^. In this respect it is interesting to note that Gruner *et al*. pointed out recently that the *a*/*b* twinning is important part of the martensite formation mechanism in adaptive 14 M Ni-Mn-Ga martensite^22^.

The unique properties of the materials resulting from the martensite structure and microstruture are also of a significant interest. The magnetic shape memory alloys exhibit a variety of magnetomechanical effects such as MFIS which are tightly linked with the high mobility of martensite twin boundaries. Particularly the Ni-Mn-Ga system shows the highest twin boundary mobility of all shape memory alloys^42^. The theoretical model by Seiner *et al*. indicates that modulation and *a*/*b* twinning can be strong factors controlling the *a*/*c* twin boundary mobility^24^. In this respect our finding that *a*/*b* twin density varies with temperature provides a unique opportunity to evaluate the effect of width of *a*/*b* twin plates on the mobility of *a*/*c* twins and of other properties of the material.

From the application point of view the magnetic shape memory alloys are expected to be used in a broad range of temperatures. Any extra phase transformation such as the one presented here and related change of properties should be well understood prior serious use of the material. Our finding gives a clear direction for further studies considering practical applications of magnetic shape memory alloys.

## Conclusion

We discovered that {110) or *a*/*b* nanotwins appear systematically in Ni_50_Mn_25+*x*_Ga_25−*x*_ single crystals with $2.6 < x < 3.5$ at low temperatures. The nanotwin size is <17 nm. The extrapolation of our results suggests that the *a*/*b* nanotwinning may also appear in stoichiometric alloy Ni_50_Mn_25_Ga_25_, which possibility has not been considered before.

The finding can have a strong impact on any study attempting to determine or refine the structure of Ni-Mn-Ga compounds, since in the case of nanotwinning the diffracted pattesrn is distorted and false crystal symmetries can emerge. For any structural study on Ni-Mn-Ga it is critically important to recognize, whether the nanotwinning occurs or not for the particular alloy under investigation.

## Methods

The single crystals of composition Ni_50_Mn_25+*x*_Ga_25−*x*_ (at. %, $2.6 < x < 3.9$) with 10 M martensite structure were obtained from Adaptamat Ltd. Samples with dimensions close to 0.9 × 2.4 × 10 mm^3^ were cut along {100} planes and electropolished. All samples exhibited about 6% magnetic field induced strain.

XRD measurements on the single crystal samples were performed using two laboratory diffractometers with parallel beam optics and Euler cradle. A high resolution measurement of 2*θ*−*ω* map was made using PANalytical Empyrean diffractometer with hybrid monochromator and Cobalt tube (*λ* = 0.17890 nm). The (400), (040), and (004) diffraction lines were measured in Bruker D8 Discover diffractometer equipped with rotating Cu anode (*λ* = 0.15406 Â nm) and cooling stage Anton Paar DCS 350. The stage temperature was varied from 350 K to 170 K.

The samples were first compressed by a few MPa along their long geometrical axis, which resulted in a uniform orientation of the *c*-axis. The resulting sample still exhibited rich internal structure with the {100) modulation twins and {110) compound *a*/*b* twins. Owing to the latter we could observe the (400) and (040) diffraction lines simultaneously for a single orientation of the sample.

The diffraction maxima of the single crystals were first located using *ω* and *χ*-scans. After that 2*θ*−*ω* scans were measured with corresponding offsets. To achieve relevant precision, the obtained diffraction profiles were evaluated by in-house software allowing advanced fitting of the peaks with Pearson VII functions^43^ corresponding to appropriate K _*α*_ doublet. The width and shape parameters of Pearson VII function were constrained to be the same in each diffractogram.

The *a*/*b* twins were directly observed in the scanning electron microscope (SEM) Tescan FERA3 using back-scattered electrons (BSE). The method and settings were the same as in ref.^26^, where detailed description can be found. The accelerating voltage was 30 kV and the BSE signal was obtained by a scintillating annular YAG single crystal detector. Owing to electron channeling the BSE can reveal the crystallographic orientation of the lattice. The small differences in the lattice orientation of *a*/*b* twins can be visible as a weak contrast, when the sample tilt is properly set. In order to better expose the observed weak contrast, gamma (contrast) and brightness were adjusted in acquired figures. The used integrated tilting stage allowed the tilt with precision of 0.05°. Cooling and heating of the sample between 223 to 333 K was achieved by the integrated Peltier element-based cooling/heating stage.

The DFT calculations were performed using the *Vienna Ab Initio Simulation Package* (VASP)^44,45^, in which the electron-ion interaction was described by projector augmented-wave potentials^46,47^. The electronic orbitals were expanded in terms of plane waves with a maximum kinetic energy of 600 eV. The exchange and correlation energy was treated in a generalized gradient approximation with parametrization proposed by Perdew, Burke, and Ernzerhof^48^. The Brillouin zone (BZ) was sampled using a Γ-point-centered 15 × 12 × *k*_*Z*_ mesh, with *k*_*Z*_ equal to 3, 2 and 1 for supercell with 40, 80 and 120 atoms, respectively. The integration over the BZ used Methfessel-Paxton smearing method^49^ with a 0.02 eV smearing width. The settings for *k*-point mesh and smearing width were obtained with the help of an adaptive smearing method^50^. The total energy was calculated with high precision by convergence to 10^−7^ eV per computational cell. Relaxation of the atomic positions and structural parameters was performed with a quasi-Newton algorithm, using the exact Hellmann-Feynman forces, and was considered to be converged after all forces dropped below 1 meV/Å.

### Data Availability

The datasets generated during and/or analysed during the current study are available from the corresponding author on reasonable request.

## Electronic supplementary material

Supplementary information
