# Supplementary material for: Low temperature a/b nanotwins in Ni50Mn25+xGa25−x Heusler alloys
Source: Sci Rep. 2018 Aug 9;8:11943. doi: 10.1038/s41598-018-30388-8 (PMC6085376; doi:10.1038/s41598-018-30388-8)
Supplement: Supplementary file 1 — Supplementary information [file 41598_2018_30388_MOESM1_ESM.pdf]

SUPPLEMENTARY INFORMATION

Low temperature *a*/*b* nanotwins in $Ni_{50}Mn_{25+x}Ga_{25-x}$ Heusler alloys

L. Straka[1,*], J. Drahokoupil[1], P. Veřtát[1], M. Zelený[2,3], J .Kopeček[1], A. Sozinov[4], and O. Heczko[1,2]

[1]*Institute of Physics, Czech Academy of Sciences, Na Slovance 2, 182 21 Prague, Czech Republic*

[2]*Faculty of Mathematics and Physics, Charles University in Prague, Ke Karlovu 5, 12116 Prague, Czech Republic*

[3]*Institute of Materials Science and Engineering, NETME Centre, Faculty of Mechanical Engineering, Brno University of Technology, Technická 2896/2, 616 69, Brno, Czech Republic*

[4]*Lappeenranta University of Technology, Material Physics Laboratory, Laitaatsillantie 3, Savonlinna, 57170, Finland*

Here we present high resolution original micrographs of a/b twins (compound {110} twins) observed by SEM using BSE and channeling contrast.

* *corresponding author. Email: ladislav.straka@fzu.cz, Phone: +420 266 052 860*

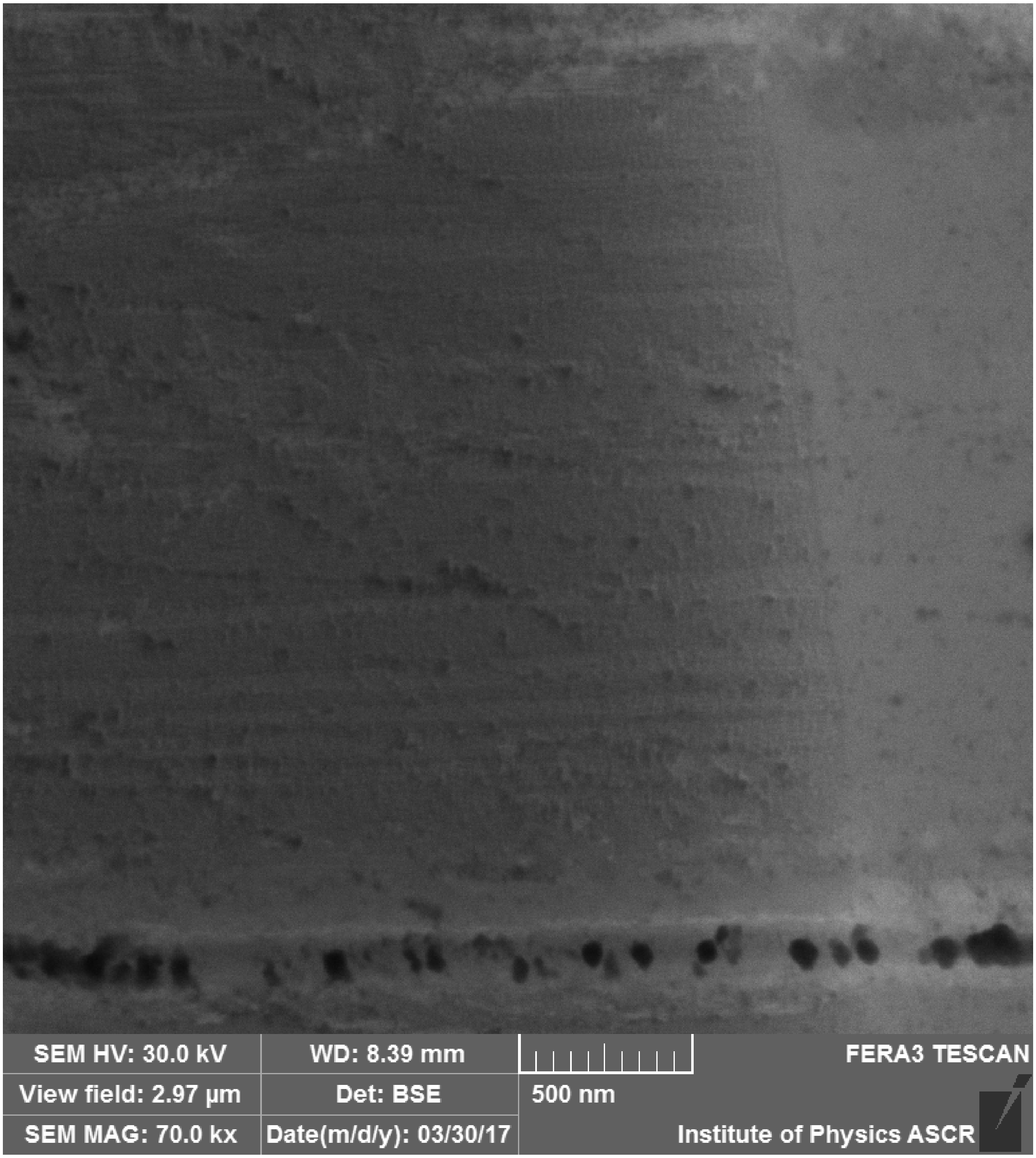

Original micrograph for Fig. 5a

Horizontal band contrast related to a/b twinning obtained using BSE in SEM in the $Ni_{50.0}Mn_{28.2}Ga_{21.8}$ single crystal (298 K).

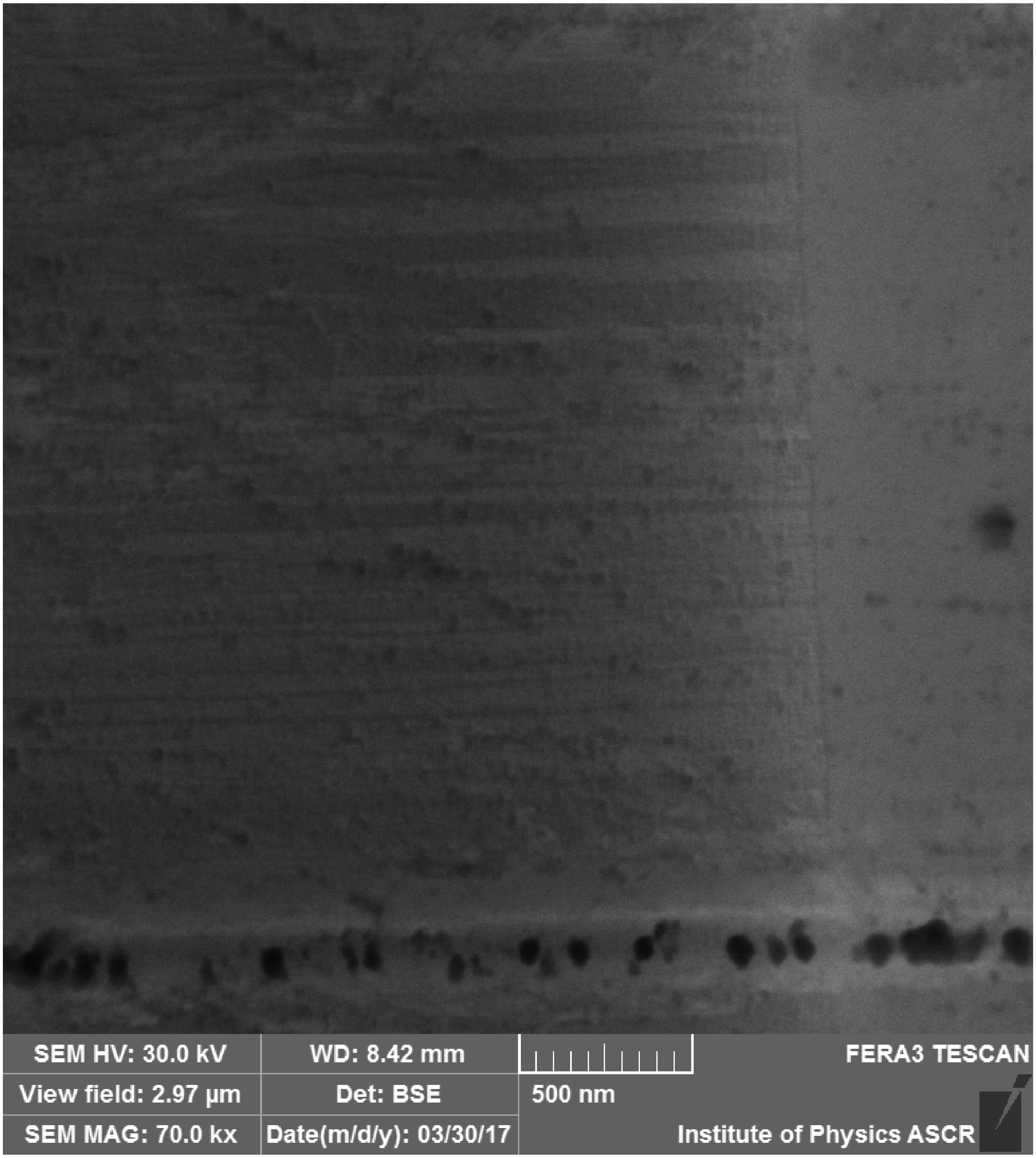

Original micrograph for Fig. 5b

Horizontal band contrast related to a/b twinning obtained using BSE in SEM in the $Ni_{50.0}Mn_{28.2}Ga_{21.8}$ single crystal (273 K).

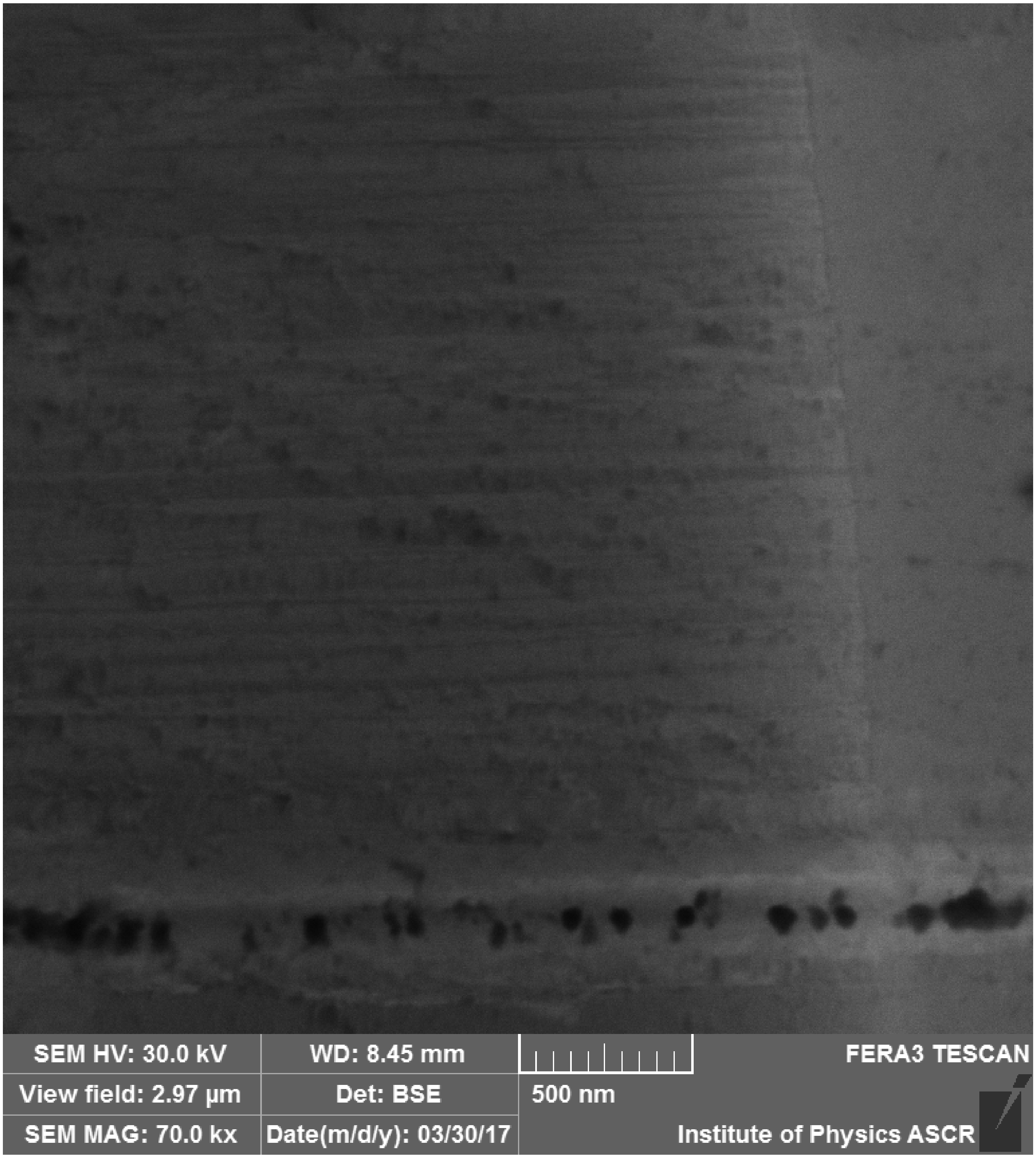

Original micrograph for Fig. 5c

Horizontal band contrast related to a/b twinning obtained using BSE in SEM in the $Ni_{50.0}Mn_{28.2}Ga_{21.8}$ single crystal (253 K).

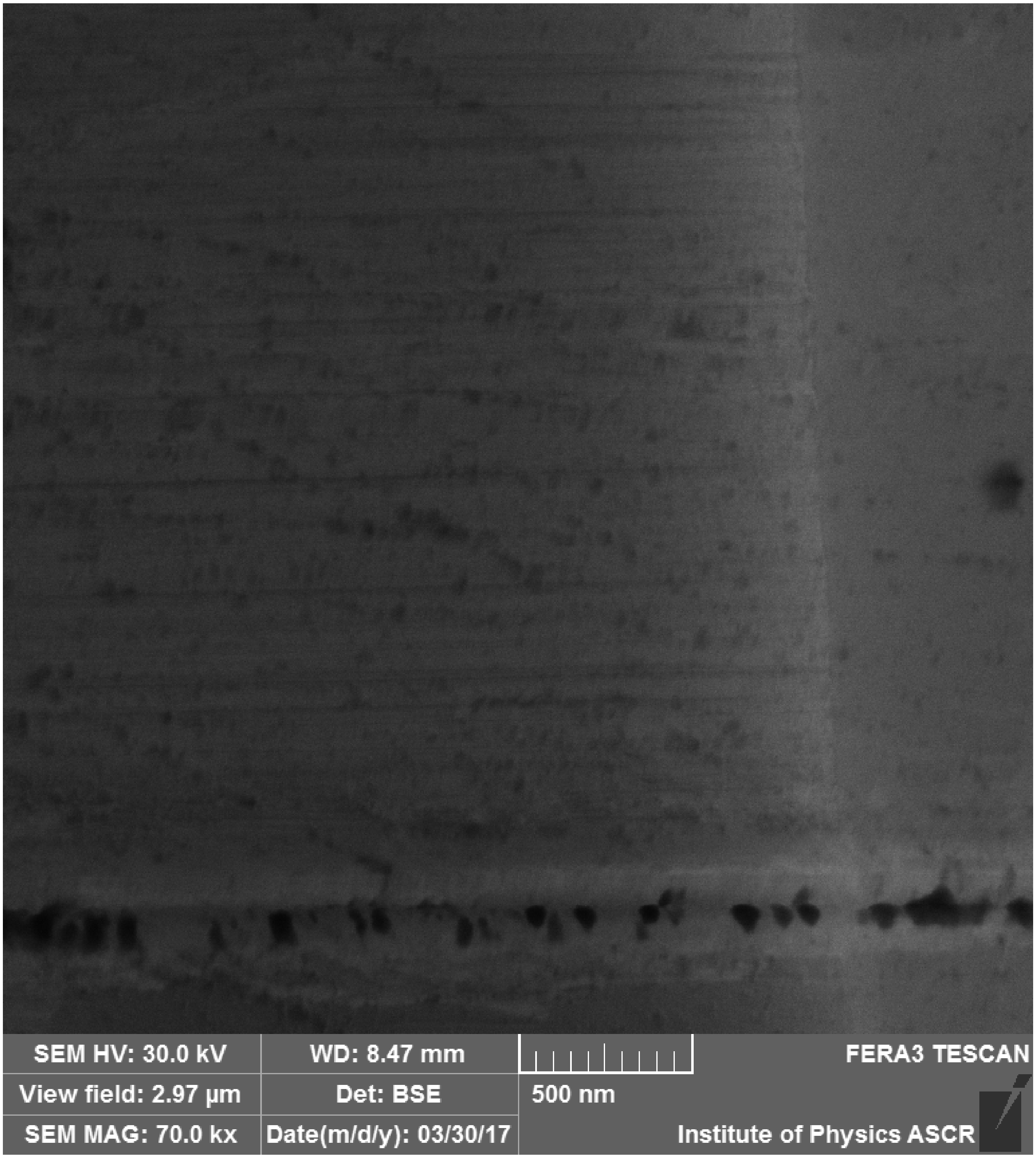

Original micrograph for Fig. 5d

Horizontal band contrast related to a/b twinning obtained using BSE in SEM in the $Ni_{50.0}Mn_{28.2}Ga_{21.8}$ single crystal (223 K).

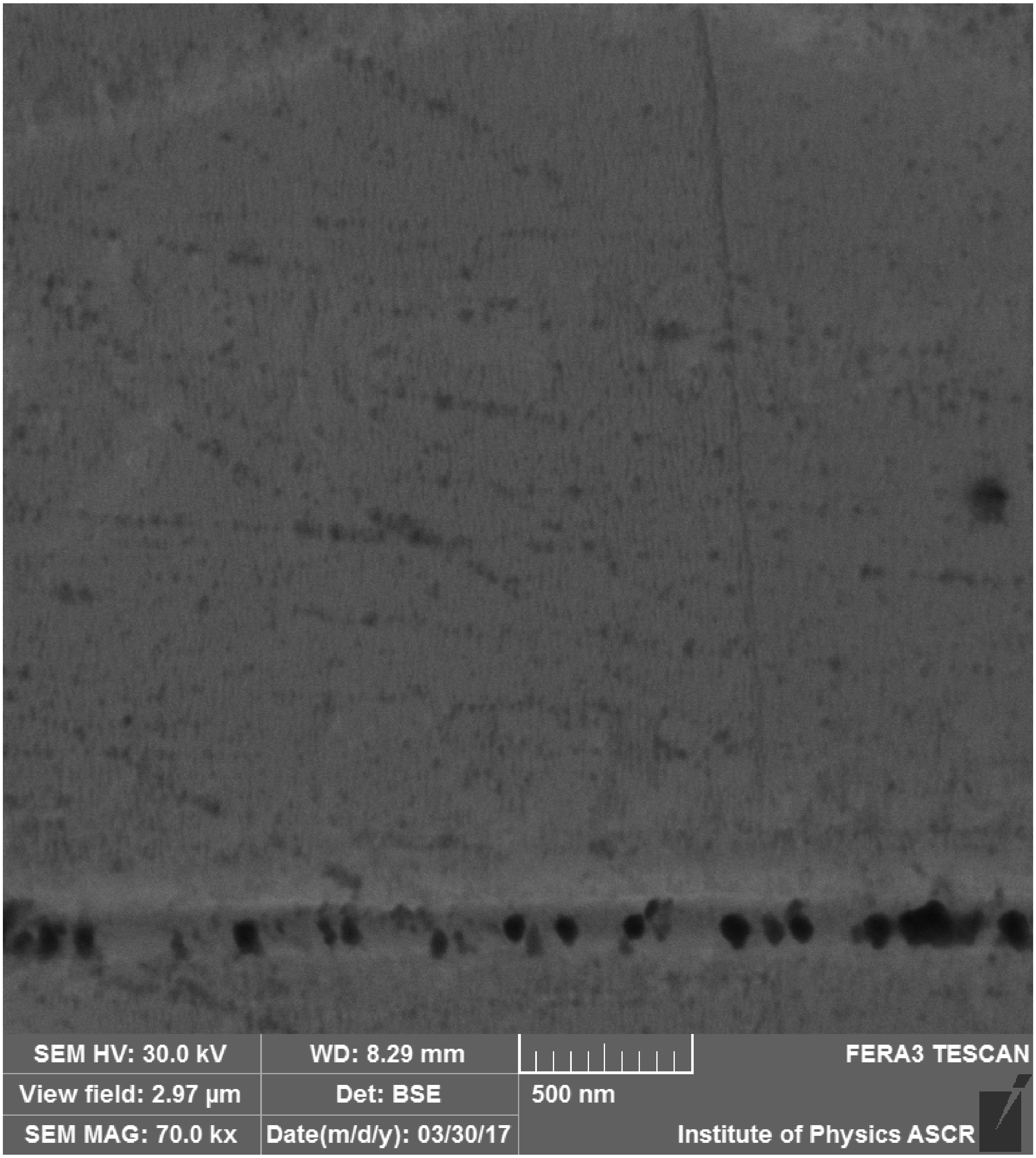

Original micrograph for Fig. 5f

Horizontal band contrast related to a/b twinning obtained using BSE in SEM in the $Ni_{50.0}Mn_{28.2}Ga_{21.8}$ single crystal (no horizontal band contrast observed in austenite, 333 K).
